# Angiotensin II Promotes Skeletal Muscle Angiogenesis Induced by Volume-Dependent Aerobic Exercise Training: Effects on miRNAs-27a/b and Oxidant–Antioxidant Balance

**DOI:** 10.3390/antiox11040651

**Published:** 2022-03-28

**Authors:** Luis Felipe Rodrigues, Bruno Rocha Avila Pelozin, Natan Daniel da Silva Junior, Ursula Paula Renó Soci, Everton Crivoi do Carmo, Glória de Fatima Alves da Mota, Victoria Cachofeiro, Vicente Lahera, Edilamar Menezes Oliveira, Tiago Fernandes

**Affiliations:** 1 Laboratory of Biochemistry and Molecular Biology of Exercise, School of Physical Education and Sport, University of Sao Paulo, Sao Paulo 05508-030, Brazil; l.rodrigues@usp.br (L.F.R.); bruno.pelozin@usp.br (B.R.A.P.); natanjr@usp.br (N.D.d.S.J.); ursula.soci@usp.br (U.P.R.S.); gfamota@usp.br (G.d.F.A.d.M.); 2 Physiotherapy Program, Ibirapuera University, Sao Paulo 04661-100, Brazil; 3 Physical Education, Senac University Center, Sao Paulo 04696-000, Brazil; evertoncrivoi@usp.br; 4 Department of Physiology, School of Medicine, Universidad Complutense de Madrid, 28040 Madrid, Spain; vcara@med.ucm.es (V.C.); vlahera@med.ucm.es (V.L.)

**Keywords:** aerobic training, microRNAs, angiogenesis, renin–angiotensin system, redox balance

## Abstract

Aerobic exercise training (ET) produces beneficial adaptations in skeletal muscles, including angiogenesis. The renin–angiotensin system (RAS) is highly involved in angiogenesis stimuli. However, the molecular mechanisms underlying capillary growth in skeletal muscle induced by aerobic ET are not completely understood. This study aimed to investigate the effects of volume-dependent aerobic ET on skeletal muscle angiogenesis involving the expression of miRNAs-27a and 27b on RAS and oxidant–antioxidant balance. Eight-week-old female Wistar rats were divided into three groups: sedentary control (SC), trained protocol 1 (P1), and trained protocol 2 (P2). P1 consisted of 60 min/day of swimming, 5×/week, for 10 weeks. P2 consisted of the same protocol as P1 until the 8th week, but in the 9th week, rats trained 2×/day, and in the 10th week, trained 3×/day. Angiogenesis and molecular analyses were performed in soleus muscle samples. Furthermore, to establish ET-induced angiogenesis through RAS, animals were treated with an AT1 receptor blocker (losartan). Aerobic ET promoted higher VO_2_ peak and exercise tolerance values. In contrast, miRNA-27a and -27b levels were reduced in both trained groups, compared with the SC group. This was in parallel with an increase in the ACE1/Ang II/VEGF axis, which led to a higher capillary-to-fiber ratio. Moreover, aerobic ET induced an antioxidant profile increasing skeletal muscle SOD2 and catalase gene expression, which was accompanied by high nitrite levels and reduced nitrotyrosine concentrations in the circulation. Additionally, losartan treatment partially re-established the miRNAs expression and the capillary-to-fiber ratio in the trained groups. In summary, aerobic ET promoted angiogenesis through the miRNA-27a/b–ACE1/Ang II/VEGF axis and improved the redox balance. Losartan treatment demonstrates the participation of RAS in ET-induced vascular growth. miRNAs and RAS components are promising potential targets to modulate angiogenesis for combating vascular diseases, as well as potential biomarkers to monitor training interventions and physical performance.

## 1. Introduction

The stimuli triggered by exercise training (ET) cause beneficial adaptations in skeletal muscles, characterized by increased mitochondrial biogenesis, improved oxidative metabolism, increased strength, reduced fatigue, and angiogenesis. However, overexercise increases the risk of cardiovascular and overuse injuries, impairing the performance and health of individuals through an increase in chronic inflammation and increased oxidative stress [1]. Therefore, the proper combination of intensity, volume, and frequency are fundamental factors for the development of physiological adaptations promoted by exercise [1,2,3]. Among these adaptations, angiogenesis stands out as one of the main markers of aerobic capacity, mainly because it favors improved peripheral perfusion, which promotes the uptake of energetic substrates and oxygen delivery. In contrast, microvascular rarefaction has been shown to be an independent risk marker for cardiovascular diseases [4,5].

Angiogenesis is known to promote the formation of new blood vessels by endothelial cells, as it is an adaptive response generated from oxygen deprivation. Various angiogenic factors have been characterized, and vascular endothelial growth factor (VEGF) is one of the main factors in vascular growth after ischemia or ET [6]. Among the systems involved in the formation of new vessels, the renin–angiotensin system (RAS) plays a prominent role in angiogenic signaling via the effector peptide angiotensin II (Ang II) through its Ang II receptor type 1 (AT1 receptor), promoting VEGF activation [7]. Angiotensin-converting enzyme (ACE1), which acts predominantly in endothelium and sarcolemma myofibers, is a central component in RAS converting Ang I to Ang II [8]. Accordingly, VEGF has been shown to increase ACE1 expression in endothelial cells, suggesting a synergistic relation between VEGF and RAS in vascular growth [9].

Studies involving treatment with captopril (ACE blocker) and losartan (AT1 receptor blocker) showed that, when these drugs are administered, short-term aerobic exercise and electrical stimulation-induced vessel formation were inhibited, confirming the participation of Ang II in skeletal muscle angiogenesis [10,11]. Furthermore, treatment with captopril or losartan blocked VEGF expression. In addition, VEGF inhibition through the monoclonal VEGF-neutralizing antibody was able to prevent exercise-induced angiogenesis [10]. Studies also show that, in a single exercise session, it was possible to observe the elevation of VEGF mRNA in skeletal muscles in both humans [12,13,14] and rats [15,16,17].

In addition to the proangiogenic effects, the acute or low-dose release of Ang II is determined by reduced apoptosis and increased recruitment of endothelial progenitor cells, which contribute to vascular repair and integrity. On the other hand, the chronic or high-dose release of Ang II promotes the exacerbated release of nicotinamide adenine dinucleotide phosphate oxidase (NADPH oxidase), a reactive oxygen species (ROS)-generating enzyme that is present in the cell membrane and is involved in cellular damage. This in turn induces apoptosis and microvascular rarefaction [7]. Activation of these redox-sensitive pathways regulates vascular growth, inflammation, and senescence. Although there is much evidence indicating the role of the Ang II/ROS axis in pathological conditions [18], there is still a lack of information regarding how Ang II exerts effects through ROS induced by different ET types and volumes.

MicroRNAs (miRNAs) are a class of highly conserved, non-coding small RNAs that regulate gene expression on the post-transcriptional level by inhibiting the translation of a protein or by promoting the degradation of mRNA [19]. Our group previously identified several miRNAs (miRNAs-208a, -208b, -143, -126, -29c, -27a, -27b, -21, -16) in the heart and skeletal muscle that are associated with metabolic, contractile, angiogenic, fibrotic, and epigenetic responses induced by aerobic ET [5,20,21,22,23,24]. In particular, we showed changes on miRNAs-27a and -27b targeting ACE1 in physiological cardiac hypertrophy induced by aerobic ET [5]. In addition, miRNAs-27b was shown to significantly promote angiogenesis [25]. However, RAS signaling-related miRNAs as skeletal muscle angiogenesis modulators that are induced by aerobic ET remain unknown.

In this study, we aimed to investigate the effects of volume-dependent aerobic ET on skeletal muscle angiogenesis involving miRNAs-27a and -27b expression, RAS, and oxidant–antioxidant balance. Our hypothesis is that aerobic ET promotes volume-dependent skeletal muscle angiogenesis, which is accompanied by a reduction in miRNAs-27a and -27b and the stimulation of the ACE1/Ang II/VEGF axis without impairing redox balance.

## 2. Materials and Methods

### 2.1. Animal Care

**Figure 1:**
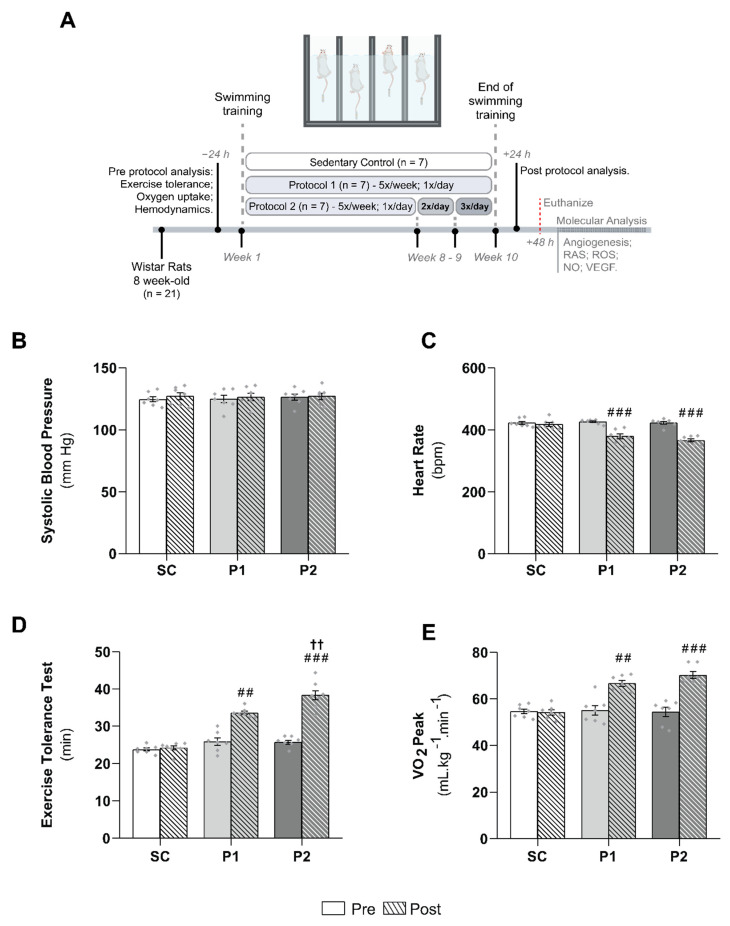
Experimental design and effect of aerobic exercise training on hemodynamic parameters and training markers: (**A**) experimental design; (**B**) systolic blood pressure; values are expressed in millimeters of mercury (mm Hg); (**C**) heart rate values, expressed in beats per minute (bpm); (**D**) exercise tolerance test duration, expressed in minute (min); (**E**) peak oxygen uptake (VO_2_ peak) values, expressed in milliliters of oxygen per kilogram of body mass per minute (mL.kg^−1^.min^−1^). Groups: SC, sedentary control (*n* = 7); P1, swimming trained following protocol 1 (*n* = 7); P2, swimming trained following protocol 2 (*n* = 7). Results are expressed as mean ± SEM. Statistical analysis: one-way ANOVA with repeated measures. Each dot indicates a biological replication. Significantly different vs. ## pre- and post-test, *p* < 0.001; ###, *p* < 0.0001; †† P1, *p* < 0.001.

All procedures and protocols used were in accordance with the local ethics committee of the University of Sao Paulo (No. 2019/01) and were conducted in accordance with the Guide for the Care and Use of Laboratory Animals, published by the US National Institutes of Health (NIH Publication No. 85–23, revised 1996). A total of 42 eight-week-old female Wistar rats (180–220 g) were used. The animals were weighed weekly and housed three to five per cage at a controlled room temperature (22 ± 2 °C) with a 12 h dark–light cycle. They were fed standard rat chow with access to water ad libitum. The rats were randomly assigned to the following experimental groups: sedentary control (SC, *n* = 7), swimming trained following protocol 1 (P1, *n* = 7), and swimming trained following protocol 2 (P2, *n* = 7), as described below and illustrated in Figure 1A. To assess the participation of RAS in skeletal muscle angiogenesis, losartan (AT1 receptor blocker) was used. The animals were administered 20 mg. kg^−1^.day^−1^ of losartan in their drinking water. Three more groups were randomly assigned, i.e., sedentary control + losartan (SC + los, *n* = 7), swimming trained following protocol 1 + losartan (P1 + los, *n* = 7), and swimming trained following protocol 2 + losartan (P2 + los, *n* = 7).

### 2.2. Exercise Training Protocol

Protocol 1 (P1) consisted of a 60 min swimming session, 5 times a week, for 10 weeks, with a 5% caudal body weight (BW) workload. In protocol 2 (P2), animals performed the same swimming-training protocol as in P1 until the end of the 8th week. Over the two following weeks, rats were trained for 60 min, twice a day in the 9th week, and three times a day in the 10th week. Every training session had a 4 h interval between sessions. Figure 1A illustrates the training protocol groups. Animals were trained in a swimming apparatus specially designed to allow individual exercise training of rats. A heating system kept the water temperature between 30 and 32 °C, and swimming sessions were carried out from 7:30 to 8:30 (P1 and P2) AM, from 12:30 to 1:30 PM (P2), and from 5:30 to 6:30 PM (P2). The aim of increasing the training volume in P2 was to increase aerobic capacity. The SC group was placed in the swimming apparatus for 10 min twice a week without a workload to control for being in the water and underwent the same procedures as the training protocol groups. These protocols were used previously in our laboratory and are effective for promoting cardiovascular adaptations [5,20,21,22,23,24,26]. After the final ET session, the animals performed an exercise tolerance test and were assessed as regards their hemodynamic parameters and peak oxygen uptake.

### 2.3. Cardiovascular Measurements

Resting arterial systolic blood pressure (SBP) and heart rate (HR) were measured in conscious rats, with the use of a computerized tail–cuff system (IITC Life Science, Los Angeles, CA, USA) [27]. Rats were acclimatized to the apparatus during daily sessions held over 4 days, 1 week before starting the experimental period. BP and HR were determined before and after the ET protocol.

### 2.4. Exercise Tolerance Test

The exercise capacity, estimated by the total running time, was evaluated with a graded treadmill exercise protocol for rats. After being adapted to treadmill exercises over 1 week (10 min of exercise per session), rats were placed in the exercise streak and allowed to acclimatize for at least 30 min. The exercise began at 6 m/min (meters per minute) with no grade and increased by 3 m/min every 3 min thereafter until exhaustion. Exhaustion was defined as the failure to maintain the current speed as previously described [28].

### 2.5. Oxygen Uptake Measurements

Oxygen consumption (VO_2_) was measured by an expired gas analysis during the graded treadmill exercise described above. Gas analysis was performed using an oxygen and carbon oxide analyzer (Sable Systems SS3, FC-10a O_2_/CO_2_ analyzer, Las Vegas, NV, USA). VO_2_ was calculated using the measured flow through the metabolic chamber, the expired fraction of effluent oxygen, and the fraction of oxygen in room air [24,28].

### 2.6. Preparation of Biological Samples

After 48 h of the experimental period, the rats were killed by decapitation after anesthesia (3–5% isoflurane by inhalation) and tissue samples were harvested, weighed, frozen, stored at −80 °C, and used for the enzyme assay, and miRNA, mRNA, and protein preparation. To separate plasma, centrifugation at 1500× *g* for 15 min using a refrigerated centrifuge at 4 °C was performed immediately after collecting blood in tubes containing heparin anticoagulant. Following centrifugation, the plasma was immediately transferred into sterile polypropylene tubes and stored at –80 °C.

### 2.7. Capillary-to-Fiber Ratio

The capillary-to-fiber ratio of the soleus muscle was evaluated after myofibrillar ATPase histochemistry reaction at pH 10.3 as previously described [29]. Briefly, the capillary-to-fiber ratio was quantified using a 10 × 10 grid optically superimposed on each of five non-overlapping fields at a 200× magnification, distributed in a random manner using a computer-assisted morphometric system (Quantimet 500, Leica, Cambridge, UK). To calculate the capillary-to-fiber ratio, the total number of capillaries was divided by the total number of fibers counted in the same field. Only vessels with a diameter less than 10 μm were counted, which largely comprised capillaries but may have also included terminal arterioles or venules. All analyses were conducted by a single observer (T.F.) blinded to rat identity.

### 2.8. Measurement of Plasmatic Nitric Oxide Concentration

Plasma NO concentration was determined by nitrite dosage using a Sievers Nitric Oxide Analyzer (NOA 280) (GE Analytical Instruments, Boulder, CO, USA). To measure nitrite, the purge vessel contained a reducing agent (1% potassium iodide in glacial acetic acid) to convert nitrite to NO. The NO produced was swept into the NOA where it reacted with ozone, forming electronically excited nitrogen dioxide; the associated emission was proportional to the amount of NO present in the sample. The amount of NO present was determined by integrating the emission signal over time and was calibrated using known amounts of nitrite as a source of NO [30].

### 2.9. Measurement of Plasmatic Nitrotyrosine

Plasma nitrotyrosine concentration was determined by Bioxytech Nitrotyrosine enzyme immunoassay, according to the manufacturer’s instructions (OxisResearch, Portland, OR, USA) [30].

### 2.10. ACE1 and ACE2 Activity Assay

ACE1 activity was determined in the soleus muscle using fluorescent substrates [5]. Frozen skeletal muscle samples were homogenized in 0.1 M Tris-HCl buffer pH 7.0, containing 50 mM NaCl, and centrifuged at 1,000×g for 10 min. The assays were performed at 37 °C in 0.1 M Tris-HCl buffer pH 7.0, containing 50 mM NaCl and 10 µM ZnCl2, and captopril 0.5 µL as an inhibitor in negative samples. The hydrolysis rate of the intramolecularly quenched fluorogenic substrate Abz-FRK-(Dnp)P-OH (10 uM) incubated with aliquots of homogenate for 30 min at 37 °C was assessed to obtain ACE enzymatic activity (420 nm λem and 320 nm λex, read in 90 cycles). ACE2 activity was determined by the same method described above. However, Abz-APK(Dnp)-OH was used as the fluorescent peptide, in 0.2 M Tris-HCl buffer, 200 mM NaCl, pH 7.5, and DX600 1 mM as the inhibitor. ACE1 and ACE2 activities are expressed as uF.min^−1^.mg^−1^ of the skeletal muscle protein concentration.

### 2.11. Measurement of Angiotensin II Levels

Soleus samples were homogenized in lysis buffer (0.1 M sodium phosphate, 0.34 M sucrose, 0.3 M NaCl), containing a mixture of protease inhibitors, and centrifuged at 10,000× *g*, at 4 °C, for 10 min. The supernatant was collected, passed through phenyl silica cartridges (Sep-Pak C18 columns; Waters, Livingston, Scotland), and the absorbed angiotensin was eluted with methanol. The eluate was dried in a vacuum centrifuge and the pellet was resuspended in EIA buffer, mixed, and centrifuged at 3000× *g* for 10 min at 4 °C. Ang II levels were determined by ELISA, according to the manufacturer’s instructions (SPI-BIO), and as previously described [31]. The protein content was determined using the Bradford method [32] using bovine serum albumin as the standard (Bio-Rad Protein Assay, Hercules, CA, USA).

### 2.12. NADPH Oxidase Activity

As was shown previously [33], NADPH oxidase activity was assessed in a soleus membrane-enriched fraction. The soleus samples (~40 mg) were homogenized in a lysis buffer (50 mM Tris·HCl, 0.1% mercaptoethanol, 10 g/ml aprotinin, 10 g/mL leupeptin, 1 mM phenylmethylsulfonyl fluoride, and 0.01 mM diethylenetriaminepentaacetic acid (DPTA), at 4 °C, pH 7.4) and were sonicated for three cycles of 10 s each at 8 W. To separate mitochondria and nuclei, the samples were centrifuged at 18,000× *g* for 15 min. To obtain a membrane-enriched fraction, the supernatants were then centrifuged at 100,000× *g* for 1 h. Membrane fractions (20 μg of protein) were incubated with DHE (50 μM), DNA (1.25 μg/mL), and NADPH (50 μM) in PBS–DTPA (final volume of 120 μL) for 30 min at 37 °C in the absence of light. Fluorescence was measured (λexc 510 nm, λem 595 nm) in a microplate reader (SpectraMax M5; Molecular Devices, San Jose, CA, USA).

### 2.13. RNA Isolation and mRNA Quantification Using Real-Time PCR

Frozen skeletal muscle samples were homogenized in Trizol, and RNA was isolated according to the manufacturer’s instructions (Invitrogen Life Technologies, Carlsbad, CA, USA. Following extraction, total RNA concentration was quantified using a NanoDrop Spectrophotometer (NanoDrop Technologies, Wilmington, DE, USA) and checked for integrity by EtBr agarose gel electrophoresis. cDNA was synthesized using reverse transcriptase at 70 °C for 10 min and incubated at 42 °C for 60 min and 95 °C for 10 min (Invitrogen Life Technologies USA) [5,28]. The mRNA expression was assessed by oligonucleotides primers (Exxtend, Brazil) for analysis of the genes AT1 (Ensembl: ENSG00000144891): (Sense: 5′-CAC AAC CCT CCC AGA AAG TG-3′; antisense:5′-AGG GCC ATT TTG TTT TTC TG-3′), AT2 (Ensembl: ENSG00000180772): (Sense: 5′-GAA CAG AAT TAC CCG TGA CC-3′; antisense: 5′- ATG AAT GCC AAC ACA ACA GC-3′), Nox2 (Ensembl: ENSG00000165168): (Sense: 5′-CCC TTT GGT ACA GCC AGT GAA GAT-3′; antisense: 5′-CAA TCC CAG CTC CCA CTA ACA TCA-3′), p22 phox (Ensembl: ENSG00000051523): (Sense: 5′-CAG CCA TCC GGG GTG AGC AGT G-3′; antisense: 5′-GGG GTT GGT AGG TGG CTG CTT GAT G-3′), p47 phox (Ensembl: ENSG00000158517): (Sense: 5′-CAT CCC TCC ATG GCC TCT GCG TC-3′; antisense: 5′-GTC AGC ACA GGA ACT CAG GCA GGA CAG-3′), SOD2 (Ensembl: ENSG00000112096): (Sense: 5′-AGC TGC ACC ACA GCA AGC AC-3′; antisense:5′-TCC ACC ACC CTT AGGGCT CA-3′), Catalase (Ensembl: ENSG00000121691): (Sense: 5′-GCG AAT GGA GAG GCA GTG TAC-3′; antisense:5′-GAG TGA CGT TGT CTT CAT TAG CAC TG-3′), and reference gene Cyclophilin (Ensembl: ENSG00000196262): (Sense: 5′-TGG CAA GCA TGT TGG GTC TTT GGG AAG-3′; antisense: 5′-GGT GAT CTT CTT GCT GCT CTG CCA TTC-3′). Real-time PCRs were run separately, and amplifications were performed with an ABI Prism 7500 Sequence Detection System (Applied Biosystems, Beverly, MA, USA) using SYBR Green PCR Master Mix (Applied Biosystems, USA). The results were quantified as Ct values, where Ct was defined as the threshold cycle of the polymerase chain reaction at which the amplified product was first detected. Each sample was analyzed in duplicate. Relative quantities of target gene expression of SC rats were compared with the P1 and P2 groups after normalization to the reference gene values (change in threshold cycle (CT)). Fold change in mRNA expression was calculated using the differences in CT values between the two samples (CT) and Equation 2^−∆∆CT^. The data are expressed in percentages related to the SC group.

### 2.14. miRNA Quantitation Using Real-Time PCR

cDNA for the miRNA analysis was synthesized from total RNA using gene-specific primers according to the TaqMan MicroRNA Assay protocol (Applied Biosystems, Beverly, MA, USA). The 15 μL reactions obtained by the TaqMan MicroRNA Reverse Transcription Kit protocol (Applied Biosystems, Beverly, MA, USA) were incubated in a thermal cycler for 30 min at 16 °C, 30 min at 42 °C, and 5 min at 85 °C. To accurately detect mature miRNAs-27a/b, the RT-qPCR was performed using the TaqMan MicroRNA Assay protocol (Applied Biosystems, Beverly, MA, USA). The 20 µl PCR included 1.33 µL of RT product, 10 µL of TaqMan Universal PCR Master Mix II (2x), 7.67 µL of nuclease-free water, 1 µL of TaqMan probe mix, and 1 µL of primers specific to either miRNA-27a-3p (Assay ID: 408, miRBase Accession Number: MIMAT0000799, (Applied Biosystems, Beverly, MA, USA) or -27b-3p (Assay ID: 409, miRBase Accession Number: MIMAT0000798, Applied Biosystems, Beverly, MA, USA). The reactions were incubated in a 96-well optical plate at 95 °C for 10 min, followed by 40 cycles of 95 °C for 15 s and 60 °C for 1 min. Samples were normalized by evaluating U6 snRNA (Assay ID 1973, Applied Biosystems, Beverly, MA, USA) [5,28].

### 2.15. Quantification of Protein Expression

The protein expression of ACE1 and ACE2 in the soleus muscles was analyzed using Western blot analysis [34]. The frozen samples were homogenized in cell lysis buffer containing 100 mM Tris-HCl, 50 mM NaCl, 1% Triton X-100, and protease inhibitor cocktail (1:100, Sigma-Aldrich, St. Louis, MO, USA). After centrifugation (10,000× *g*, at 4 °C, for 10 min), the pellet was discarded, and the samples were subjected to (Laemmli 1:1, Sigma-Aldrich, St. Louis, MO, USA) SDS-PAGE in 10% polyacrylamide gels. Equal loading of samples (30 µg) was applied for electrophoresis, and proteins were electrotransferred to a nitrocellulose membrane (BioRad Biosciences, California, USA). The blot membrane was then incubated in a blocking buffer (5% BSA, 10 mM Tris-HCl, pH 7.6, 150 mM NaCl, and 0.1% Tween 20) for 2 h at room temperature and then incubated overnight at 4 °C with mouse anti-ACE 1 (ab11734, 1:100, Abcam, Waltham, MA, USA) and rabbit anti-ACE2 (sc-20998 1:200, Santa Cruz, CA, USA). The binding of the primary antibody was detected with the use of a peroxidase-conjugated secondary antibody, and enhanced chemiluminescence reagents (Amersham Biosciences, Bath, UK) were used to visualize the autoradiography. Quantification blot analyses were performed using ImageJ software (National Institute of Health, Bethesda, MD, USA), normalized to relative changes in mouse anti-α tubulin (1:1000, Santa Cruz, CA, USA).

### 2.16. Statistical Analysis

The results are expressed as mean ± standard error of the mean (SEM). Statical analyses were performed using GraphPad Software, La Jolla, CA, USA; and *p* < 0.05 was adopted as statical significant. Data normality was assessed using a Shapiro–Wilk’s test. Statistical analysis was performed using one-way analysis of variance (ANOVA) to compare the values from the three groups (SC, P1, and P2) and Tukey’s post hoc test was applied for differences. To compare pre- and post-groups, we used one-way (ANOVA) with repeated measures. To compare losartan treatment group values (SC, SC + los, P1, P1 + los, P2, and P2 + los), we used a two-way analysis of variance (ANOVA), and Tukey’s post hoc test was applied for differences. The correlation analysis was performed using Pearson’s method.

## 3. Results

### 3.1. Hemodynamics Parameters and Aerobic Training Markers

Figure 1 summarizes the pre- and post-experimental protocol hemodynamic parameters and the aerobic ET markers, such as the duration of the exercise tolerance test and the VO_2_ peak in the groups. There were no differences in blood pressure (Figure 1B), heart rate (Figure 1C), exercise tolerance (Figure 1D), or VO_2_ peak (Figure 1E) among all groups in the pre-experimental protocol, or in systolic blood pressure post-protocol. However, heart rate decreased significantly after 10 weeks of swimming training in P1 (Pre: 427 ± 3 bpm; Post: 380 ± 8 bpm, *p* < 0.0001) and P2 (Pre: 423 ± 5 bpm; Post: 366 ± 5 bpm, *p* < 0.0001). Furthermore, the aerobic ET protocol increased VO_2_ peak values (P1: Pre: 55 ± 2 mL.kg ^−1^.min ^−1^; Post: 67 ± 1 mL.kg ^−1^.min, *p* < 0.001; P2: Pre: 54 ± 2 mL.kg ^−1^.min ^−1^; Post: 70 ± 2 mL.kg ^−1^.min, *p* < 0.0001) and exercise tolerance test duration (P1: Pre: 26 ± 1 min; Post: 34 ± 1 min, *p* < 0.001; P2: Pre: 26 ± 1 min; Post: 38 ± 1 min, *p* < 0.0001) in both trained groups. Moreover, the exercise tolerance test was significantly different between P1 and P2 groups (*p* < 0.001).

### 3.2. miRNAs-27a and -27b Expression and ACE1 Target Gene Levels

**Figure 2:**
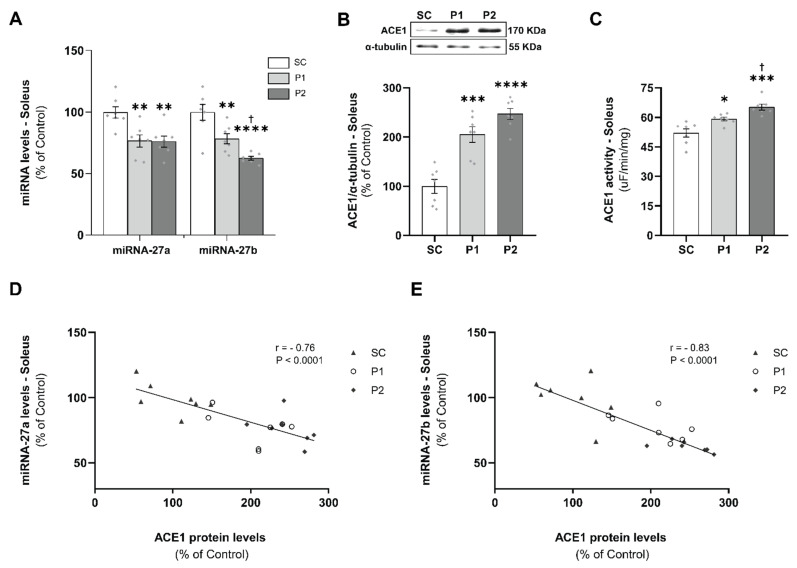
Aerobic exercise training reduced miRNAs-27a and -27b levels in a volume-dependent manner and increased angiotensin-converting enzyme 1 (ACE1) protein levels and activity in the soleus muscle: (**A**) miRNAs-27a and -27b expression; (**B**) ACE1 protein expression normalized for α-tubulin accompanied by its representative blot; (**C**) ACE1 activity. Negative correlation between the miRNA-27a and -27b expression and the ACE 1 protein levels (**D**) and (**E**), respectively. Groups: SC, sedentary control (*n* = 7); P1, swimming trained following protocol 1 (*n* = 7); P2, swimming trained following protocol 2 (*n* = 7). Each dot indicates a biological replication. Results are expressed as mean ± SEM. Statistical analysis: one-way ANOVA; correlation analysis was performed using Pearson’s method; significantly different vs. * SC, *p* < 0.05; ** SC, *p* < 0.01; *** SC, *p* < 0.001; **** SC, *p* < 0.0001; † P1, *p* < 0.05.

**Figure 3:**
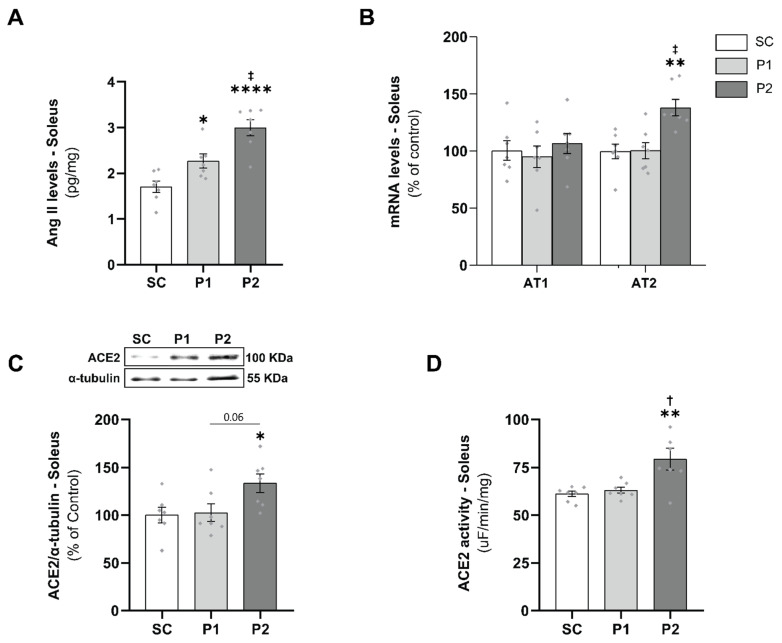
Increased Angiotensin II (Ang II), AT2 receptor, and ACE2 levels and activity in a training volume-dependent manner: (**A**) Ang II levels in the soleus muscle; (**B**) AT1 and AT2 receptors mRNA levels; (**C**) angiotensin-converting enzyme 2 (ACE2) protein expression normalized for α-tubulin accompanied by its representative blot; (**D**) ACE2 activity. Groups: SC, sedentary control (*n* = 7); P1, swimming trained following protocol 1 (*n* = 7); P2, swimming trained following protocol 2 (*n* = 7). Each dot indicates a biological replication. Results are expressed as mean ± SEM. Statistical analysis: one-way ANOVA; significantly different vs. * SC, *p* < 0.05; ** SC, *p* < 0.01; **** SC, *p* < 0.0001; † P1, *p* < 0.05; ‡ P1, *p* < 0.01.

To confirm the expression of miRNAs that target ACE1 in the soleus, miRNAs-27a and -27b were quantified by RT-qPCR. As shown in Figure 2A, the relative expression of miRNA-27a and -27b were 23% and 22% lower, respectively, in P1 (miRNA-27a: 76 ± 5%, *p* < 0.01; -27b: 78 ± 4%, *p* < 0.01), and 24% and 37% lower, respectively, in P2 (27a: 76 ± 5%, *p* < 0.01; 27b: 62 ± 2%, *p* < 0.0001), as compared with the SC groups (miRNA-27a: 100 ± 5%; -27b: 100 ± 6). In addition, miRNA-27b expression was significantly different between P1 and P2 groups (*p* < 0.05). The soleus ACE1 protein levels (Figure 3B) were increased in P1 (205 ± 16%, *p* < 0.001) and P2 (247 ± 11%, *p* < 0.0001), compared with the SC group (100 ± 14%). Interestingly, ACE1 soleus activity (Figure 3C) was increased in P1 (59 ± 1 uF/min/mg, *p* < 0.05) and P2 (65 ± 1 uF/min/mg, *p* < 0.001), compared with the SC group (52 ± 2 uF/min/mg), in a training volume-dependent manner, reaching higher values in the P2 group than in the P1 group (*p* < 0.05). Furthermore, significant negative correlations were shown between miRNAs-27a (Figure 3D) and -27b (Figure 3E) expression and the target ACE1 proteins levels in the soleus samples (miRNA-27a: r = −0.76, *p* < 0.0001; -27b: r = −0.83, *p* < 0.0001).

### 3.3. Skeletal Muscle RAS Analysis

To demonstrate whether aerobic ET modulates skeletal muscle RAS, we evaluated Ang II, AT1, and AT2 receptors and ACE2 levels. Ang II levels in the soleus muscle increased in P1 (2.3 ± 0.2 pg/mg, *p* < 0.05) and P2 (3.0 ± 0.2 pg/mg, *p* < 0.0001) in a training volume-dependent manner when compared with the SC (1.7 ± 0.1 pg/mg) and P1 groups (*p* < 0.01) (Figure 3A). AT1 and AT2 receptor mRNA levels did not change in P1, but the P2 group showed a significant increase in the AT2 receptor (138 ± 7%), compared with the SC (100 ± 6%, *p* < 0.01) and P1 groups (100 ± 7%, *p* < 0.01) (Figure 3B). Moreover, aerobic ET increased soleus ACE2 protein levels (Figure 3C) and enzyme activity (Figure 3D) in the P2 group (134 ± 7%; 79 ± 6%, respectively), compared with the SC (100 ± 8%, *p* < 0.05; 61 ± 1%, *p* < 0.01, respectively) and P1 groups (103 ± 9%, *p* = 0.06; 63 ± 2%, *p* < 0.05, respectively).

### 3.4. Exercise Training-Induced VEGF Levels and Capillary-to-Fiber Ratio

**Figure 4:**
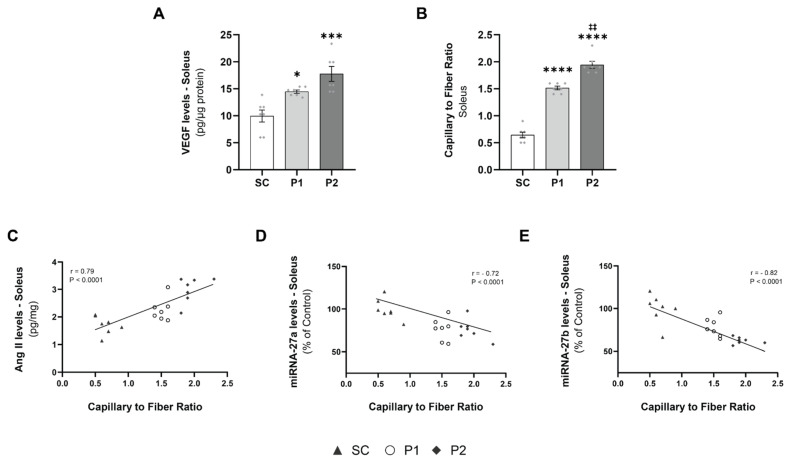
Increased vascular endothelial growth factor (VEGF) promotes a greater proportion of capillary-to-fiber ratio correlated to Angiotensin II (Ang II) and miRNAs levels: (**A**) VEGF expression levels; (**B**) capillary-to-fiber ratio; (**C**) positive correlation between angiotensin II levels and the capillary-to-fiber ratio; (**D**) negative correlation between the miRNA-27a expression and the capillary-to-fiber ratio; (**E**) negative correlation between the miRNA-27b expression and the capillary-to-fiber ratio. Groups: SC, sedentary control (*n* = 7); P1, swimming trained following protocol 1 (*n* = 7); P2, swimming trained following protocol 2 (*n* = 7). Each dot indicates a biological replication. Results are expressed as mean ± SEM. Statistical analysis: one-way ANOVA; correlation analysis was performed using Pearson’s method; significantly different vs. * SC, *p* < 0.05; *** SC, *p* < 0.001; **** SC *p* < 0.0001; ‡‡ P1, *p* < 0.0001.

To investigate aerobic ET-induced skeletal muscle angiogenesis, we evaluated the VEGF levels and the capillary-to-fiber ratio in the soleus muscle samples. VEGF levels in the soleus were increased in P1 (15 ± 1 pg/μg protein, *p* < 0.05) and P2 (17 ± 1 pg/μg protein, *p* < 0.001), compared with the SC group (10 ± 1 pg/μg protein) (Figure 4A). The capillary-to-fiber ratio in the soleus muscle exhibited an increase in angiogenesis in the trained groups in a volume-dependent manner (*p* < 0.0001), with a greater ratio in P1 (1.51 ± 0.03, *p* < 0.0001) and P2 (1.94 ± 0.06, *p* < 0.0001), compared with the SC group (0.64 ± 0.05) (Figure 4A). Moreover, Ang II levels showed a high positive correlation with the capillary-to-fiber ratio (r = 0.79, *p* < 0.0001) (Figure 4C). In contrast, miRNAs-27a (Figure 4D) and -27b (Figure 4E) expression had a significant negative correlation with capillary-to-fiber ratio (r = -0.72, *p* < 0.0001; r = 0.82, *p* < 0.0001, respectively).

### 3.5. Aerobic Exercise Training Induces Antioxidant Defense

**Figure 5:**
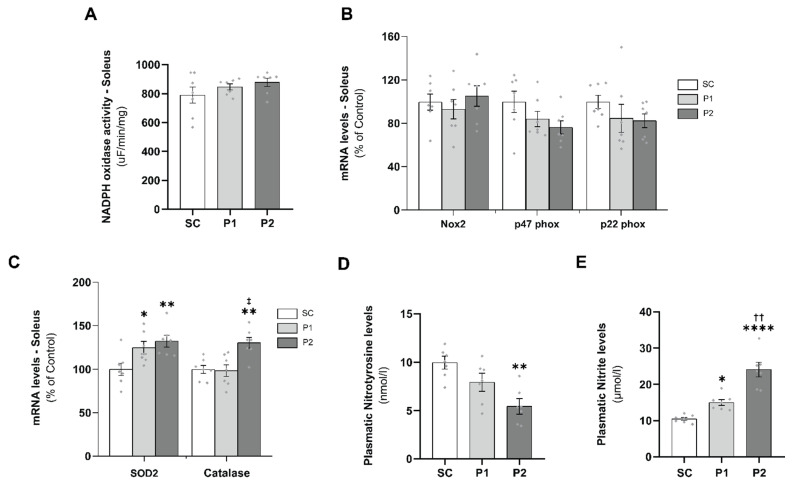
Aerobic exercise training induces antioxidant defense and redox balance: (**A**) NADPH oxidase activity in the muscle soleus; (**B**) Nox2, p47 phox, and p22 phox mRNA levels; (**C**) superoxide dismutase 2 (SOD2) and catalase mRNA levels; (**D**) plasmatic nitrotyrosine levels; (**E**) plasma nitrite levels. Groups: SC, sedentary control (*n* = 7); P1, swimming trained following protocol 1 (*n* = 7); P2, swimming trained following protocol 2 (*n* = 7). Each dot indicates a biological replication. Results are expressed as mean ± SEM. Statistical analysis: one-way ANOVA; significantly different vs. * SC, *p* < 0.05; ** SC, *p* < 0.01; **** SC, *p* < 0.0001; ‡ P1, *p* < 0.01; †† P1, *p* < 0.001.

A proper balance between pro-oxidant and antioxidant defense is necessary for physiological redox balance and to prevent the occurrence of oxidative alterations in muscle tissue. To evaluate ROS production in the soleus muscle, NADPH oxidase activity and Nox2, p47 phox, and p22 phox mRNA levels were measured. After 10 weeks of aerobic ET, NADPH oxidase activity (Figure 5A) and Nox2, p47 phox, and p22 phox mRNAs levels (Figure 5B) did not change in either trained group, compared with the SC group. Conversely, aerobic ET induced the expression of genes involved in the neutralization of reactive species such as superoxide dismutase 2 (SOD2) and catalase. SOD2 mRNA levels increased in P1 (125 ± 7%, *p* < 0.05) and P2 (132 ± 7%, *p* < 0.01), compared with the SC group (100 ± 7%), and catalase mRNAs levels increased significantly in the P2 group (130 ± 6%), compared with the SC (100 ± 5%, *p* < 0.01) and P1 groups (98 ± 7%, *p* < 0.01) (Figure 5C). Moreover, plasmatic nitrotyrosine levels, a nitro-oxidative stress marker, were reduced significantly in P2 (5.4 ± 0.8 nmol/L, *p* < 0.01) but not in P1 (7.9 ± 0.9 nmol/L, *p* = NS), compared with the SC group (10 ± 0.7 nmol/L) (Figure 5D). Corroborating this, plasmatic nitrite levels were increased in P1 (15.0 ± 0.8 μmol/L) and P2 (24.1 ± 2.0 μmol/L), compared with the SC group (10.5 ± 0.4 μmol/L) (Figure 5E).

### 3.6. Losartan Treatment Prevents Aerobic Exercise Training-Induced Skeletal Muscle Angiogenesis

**Figure 6:**
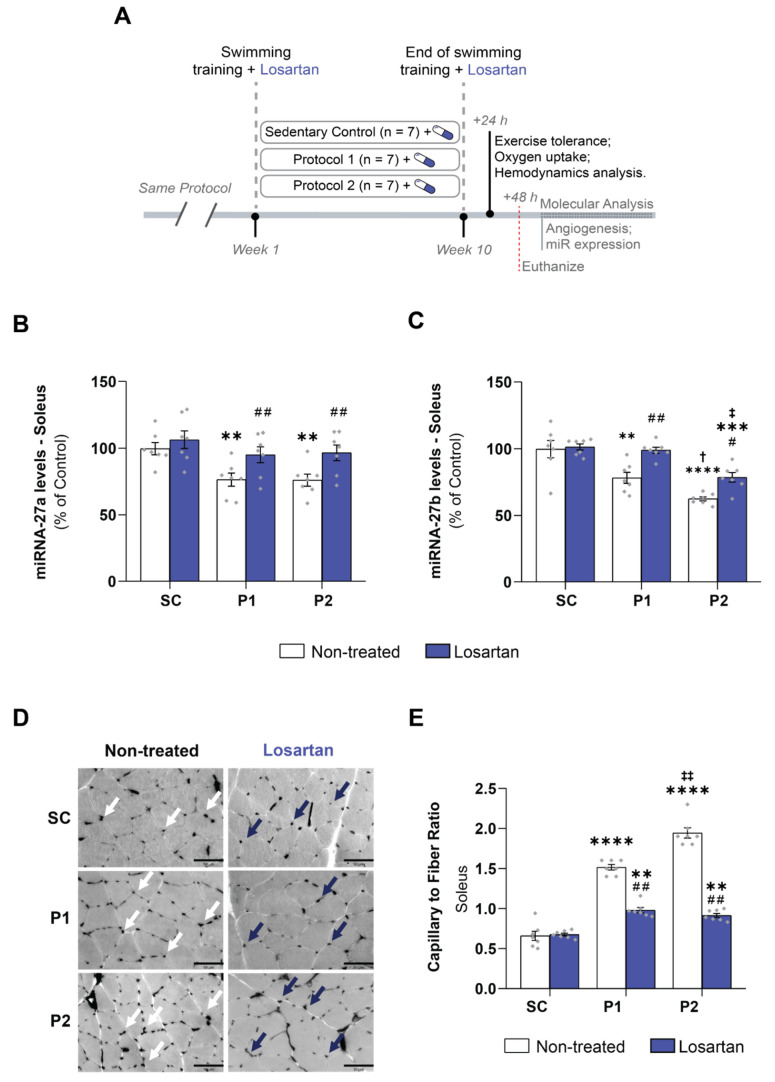
Losartan treatment prevents aerobic exercise training-induced skeletal muscle angiogenesis: (**A**) losartan treatment experimental protocol design; (**B**) miRNA-27a levels; (**C**) miRNA-27b levels; (**D**) representative figure of capillary-to-fiber ratio; (**E**) capillary-to-fiber ratio. Scale bar: 50 μm. Groups: SC, sedentary control (*n* = 7); P1, swimming trained following protocol 1 (*n* = 7); P2, swimming trained following protocol 2 (*n* = 7). Results are expressed as mean ± SEM. Statistical analysis: two-way ANOVA and Tukey’s post hoc test. Each dot indicates a biological replication. Significantly different vs. # non-treated and losartan groups, *p* < 0.01; ##, *p* < 0.001; ** SC, *p* < 0.01; *** SC, *p* < 0.001; **** SC *p* < 0.0001; † P1, *p* < 0.05; ‡ P1, *p* < 0.01; ‡‡ P1, *p* < 0.001.

To establish a relationship between skeletal muscle angiogenesis and Ang II levels induced by aerobic ET, a new group of 21 animals was trained with the same training protocols as described at the beginning of the study (Figure 6A). Moreover, they were treated with losartan, a well-known AT1 receptor blocker. The losartan treatment did not change miRNA-27a (Figure 6B) and -27b (Figure 6C) expression in the SC + los groups, compared with the non-treated animals. In the P1 groups, the losartan treatment re-established the miRNA-27a (95 ± 6%, *p* < 0.001) and -27b (99 ± 2%, *p* < 0.001) levels, compared with both the non-treated animals and the SC + los group. A similar result was observed in the P2 + los group as regards miRNA-27a (97 ± 2%, *p* < 0.001) levels, compared with the SC + los group values. However, the miRNA-27b levels were partially rescued in the P2 + los group (79 ± 2%), compared with the non-treated (*p* < 0.01), SC + los (*p* < 0.001), and P1 + los (*p* < 0.01) groups. In this way, the capillary-to-fiber ratio was partially reduced in both losartan trained groups, compared with the non-treated group (*p* < 0.01) but still exhibited significantly higher values as compared with the control-treated group (SC + los: 0.68 ± 0.02; P1 + los: 0.98 ± 0.03, *p* < 0.01; P2 + los: 0.91 ± 0.03 *p* < 0.01) (Figure 6E). These changes are shown in Figure 6D.

## 4. Discussion

Aerobic ET, if performed correctly, promotes several adaptations that generate positive responses for practitioners. This can improve performance and be used as a non-pharmacological therapy for patients with diseases. Therefore, the proper intensity, frequency, and duration of exercise are of great importance for the promotion of these beneficial responses, which include resting bradycardia, increased VO_2_ peak, increased mitochondrial biogenesis, improved oxidative metabolism, reduced fatigue, angiogenesis, improved quality of life, and reduced mortality [1,2,5,35]. In fact, our data showed that both training volumes caused endurance performance improvements, characterized by increases in exercise tolerance and oxygen consumption, followed by resting bradycardia, confirming the effectiveness of the training protocols. In addition, aerobic ET-induced angiogenesis is an adaptive physiologic response of the skeletal muscle that contributes to an improved aerobic capacity [10,12]. These structural changes in the skeletal muscle were accompanied by reciprocal regulation of miRNAs-27a and -27b in response to aerobic ET. We also observed downregulation of miRNA-27a and -27b, while the expression of its target gene ACE1 was increased by aerobic ET in a volume-dependent manner, which may contribute to Ang II/AT1 receptor/VEGF axis activation. This vascular growth signal was accompanied by a redox state balance, without altering reactive oxygen species, and an improvement in the antioxidant defense, contributing to a greater formation of new blood vessels and oxygen delivery. In contrast, treatment with losartan blocked the effects of miRNAs-27a and -27b expression and angiogenesis induced by volume-dependent aerobic ET, indicating the participation of Ang II in skeletal muscle angiogenesis.

Among all the aerobic ET-induced adaptations, angiogenesis stands out as a main adaptation in the skeletal muscle, enhancing the supply of oxygen and energetic substrates during exercise [4,10,36]. Our data showed that angiogenesis was volume-dependent in the swimming training. ET-induced angiogenesis can be described by local hypoxia, leading to VEGF activation. In contrast, hypoxia is difficult to demonstrate in muscles during endurance exercise [37]. Regardless of the stimulus, studies show that VEGF is a potent vascular growth factor induced by ET [38], in both animals [10,39] and humans [40], and is important for both its angiogenic effect and its antiapoptotic effect [41]. Thus, how ET induces VEGF and mediates exercise-induced angiogenesis remains unclear.

Conversely, experimental evidence suggests that microvascular rarefaction, which is manifested as an increase in the intercapillary distance, is responsible for non-uniform tissue perfusion, indicating poorly oxygenated areas with reduced local metabolic activity [42]. Interestingly, Debbabi [43] showed that the Framingham score for cardiovascular risk was negatively correlated with capillary density in hypertensive individuals, indicating the need for an adequate microvascular structure to maintain body homeostasis. Therefore, it is important to explore the mechanisms involved in angiogenesis as this may help to uncover new and effective strategies for vascular injuries and physical performance.

miRNAs are recognized as a major regulatory network governing gene expression in several physiological processes, interfering at the post-transcriptional levels of thousands of genes and hundreds of biological pathways [19,44]. miRNAs can also be used as biological markers and have been previously used to monitor exercise interventions [45,46,47]. Analyses of endurance athletes exposed to long training cycles show the miRNA regulation of relevant pathways in order to adapt to ET, including hypertrophy, angiogenesis, mitochondrial biogenesis, and improved oxidative metabolism [47,48]. Nevertheless, miRNAs play key roles in angiogenesis by regulating proliferation, differentiation, apoptosis, migration, and tube formation, which are indispensable for several physiological and pathological processes [49]. Emerging studies demonstrate that the dysregulation of miRNAs expression may lead to abnormal angiogenesis, which is a common feature of angiogenesis-related diseases [50]. In this way, proangiogenic therapy with miRNAs may contribute to vascular disease treatments [49] and improve aerobic capacity in endurance sports [45,46,47].

Our group was the first to identify a miRNA signature in cardiac hypertrophy induced by aerobic ET [5,20,21,22]. Similarly, we reported that the levels of endothelial-specific miRNA-126 contributed synergically to cardiac angiogenesis induced by high-volume ET [20]. Furthermore, it was shown that swimming ET upregulated miRNA-126, inducing skeletal muscle angiogenesis in obesity [24]. Accordingly, aerobic ET rescued cardiac miRNA-16 levels preventing microvascular rarefaction in obese rats [23]. Interestingly, in the present study, the downregulation of miRNAs-27a and -27b expression was significantly related to ET-induced skeletal muscle angiogenesis, indicating that these miRNAs may be regulators of vascular growth. Recently, studies showed that miRNA-27b may develop different angiogenic roles depending on the type of pathology. miRNA-27b was shown to improve angiogenesis in impaired bone marrow-derived angiogenic cells in vitro and in vivo in type 2 diabetic mice via directly inhibiting the expression of thrombospondin-1, p66shc-dependent mitochondrial oxidative stress, and Semaphorin6A [51]. In a mouse model of myocardial infarction, the authors showed that miRNA-27b mimic had overall beneficial effects, including increased vascularization, decreased fibrosis, and increased ejection fraction, at least in part due to the decreased expression of delta-like ligand 4 (Dll4), peroxisome proliferator-activated receptor γ (PPARγ), and IL-10 [25]. Conversely, miRNA-27b also represses angiogenesis. Overexpression of miRNA-27b inhibited the proliferation, migration, and tube formation of HUVECs and decreased angiogenesis in colorectal cancer and gastric cancer via inhibiting VEGFC/VEGFR2 signaling [52]. Moreover, a recent study indicated that miRNA-27b effectively repressed migration and tube formation of ovarian cancer cells, and angiogenesis in vivo by inhibiting VE-cadherin [53]. Previous results regarding miRNA-27b expression in pathological conditions remain controversial, and its ET-induced angiogenic effects remain unknown. Our study demonstrates a decrease in skeletal muscle miRNA-27b expression in a volume-dependent manner in swimming-trained rats.

miRNAs-27a and -27b have been shown to target ACE1 [54], a central component of RAS, by converting Ang I to Ang II. In our previous study, we showed that increased cardiac miRNA-27a and -27b levels inhibited ACE1 expression in trained rats [5], i.e., the increased expression of miRNAs reflected the decreased expression of target genes. These findings were, at least in part, related to physiological cardiac hypertrophy altering the expression of specific miRNAs targeting RAS genes. Our study showed a decrease in skeletal muscle miRNA-27a and -27b expression and a synergistic upregulation of the ACE1 target gene induced by aerobic ET. The increase in ACE1 expression and activity favored the increased formation of Ang II, which was dependent on the increase in exercise volume. Ang II has also been implicated in the formation of new vessels in vitro [55] and in the physiological angiogenesis induced by exercise [10] or electrical stimulation [11]. In fact, both captopril (ACE blocker) and losartan (AT1 receptor blocker) treatments have been shown to be effective in inhibiting angiogenesis induced by short-term exercise training [10] and electrical stimulation [11]. Studies show that Ang II can induce VEGF expression via the AT1 receptor [10,11,55]. We identified one pathway involving the ACE1/Ang II/AT1 receptor axis and VEGF expression in aerobic ET-stimulated skeletal muscle angiogenesis. The results from the present study confirm that RAS via losartan treatment prevented ET-induced angiogenic effects. Although the angiogenic response induced by volume-dependent aerobic ET was still present in the soleus muscle of treated rats, we observed that a drug-induced Ang II type I receptor blockade significantly attenuated the increases in the capillary-to-fiber ratio when we compared the differences between the trained and sedentary losartan-treated groups. These results suggest that other angiogenic factors resulting from aerobic ET may be involved in this process. Taken together, our study is the first to document increases in ACE1, Ang II, and VEGF levels related to skeletal muscle angiogenesis induced by long-term exercise and dependent on the increase in exercise volume.

In addition to the results observed in the classic RAS axis, alterations in the expression and activity of ACE2 in the skeletal muscle were found in the P2 group subjected to the highest volume of ET. Previous findings suggest that ACE2 maintains the important balance between Ang II and Ang (1–7) due to the fact that ACE2 cleaves Ang II to Ang (1–7), improving vasodilation and skeletal muscle metabolism [56]. In addition, the ACE2-mediated signaling pathway has antifibrotic and antiapoptotic properties [57]. Studies show that ET activates mechanisms that promote the vasoprotective and cardioprotective ACE2/Ang-(1–7) pathway [5,34]. In fact, we previously showed that aerobic ET reduced plasma Ang II concentration and increased the Ang (1–7)/Ang II ratio in the skeletal muscle of heart failure rats [34]. Thus, the high ACE2 levels in the group subjected to the highest training volume may explain the higher aerobic capacity observed in this group. This is likely because it induces greater vasodilation and vessel integrity, mediating the release of different vasoactive factors such as nitric oxide, prostaglandins, and bradykinin [58]. Accordingly, our results show higher plasma NO levels in the P2 group, concomitant with lower systemic ROS production as assessed by nitrotyrosine levels, which favored better aerobic performance.

Studies show that there is a negative correlation between molecular ACE2 levels and a higher fatality from SARS-CoV-2 [59]. In summary, after the initial entry of SARS-CoV-2 through ACE2, a consecutive reduction in ACE2 expression occurs, followed by the overactivation of the Ang II/AT1 Receptor axis, thus generating damage and disorders in multiple organs. ET promotes an increase in ACE2. This increases the availability of circulating Ang 1–7 by activating the ACE2/Ang 1–7/Mas receptor axis, which in turn induces anti-inflammatory and antifibrotic effects, thus improving the clinical results of these patients [60,61,62,63]. Studies reported that the use of ACE1 inhibitors/angiotensin-receptor blockers in hypertensive patients with COVID-19 was associated with a lower risk of mortality than those in hypertensive patients who do not use these agents, thus highlighting the safety of these drugs [63,64].

RAS overactivity, as found in chronic diseases, plays a role in skeletal myopathy, including ROS generation, atrophy, and apoptosis [65,66]. Increased ROS production in the skeletal muscle is a hallmark of chronic diseases and contributes to increased muscle disorders involving cachexia and microvascular rarefaction [67]. In addition, Ang II-induced hypertension was associated with increased vascular superoxide production via NADPH oxidase activation and endothelial dysfunction [68]. Under physiological conditions, ROS production is tightly controlled, and ROS play important roles as signaling molecules in the control of vascular tone and remodeling [18]. In this way, regular ET can benefit health by enhancing antioxidant defenses, whereas overexercise can generate excessive ROS, leading to skeletal muscle dysfunction [69]. Our results show that there was no change in the production of skeletal muscle ROS regardless of the training volume; however, higher levels of antioxidant enzymes were observed, which can contribute to an increase in the availability of muscle NO in the face of greater energy demands and oxygen supply to the exercised muscle.

In this regard, elucidating the effects of aerobic ET on the miRNAs-27/ACE1/Ang II/VEGF signaling axis would provide novel insights into how ET and different exercise volumes regulate skeletal muscle angiogenesis and aerobic capacity. Thus, ET-mediated angiogenic factors may be used as new alternative treatment strategies for vascular disorder detection, diagnosis, and prognosis, highlighting the importance of a healthy microcirculation.

## 5. Conclusions

**Figure 7:**
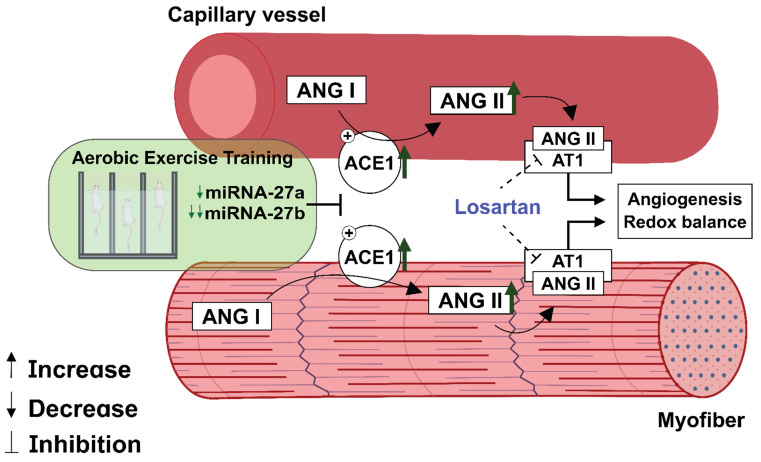
Aerobic exercise training induces miRNAs-27a and -27b inhibition alongside an increase in the ACE1/Ang II/VEGF axis, which leads to skeletal muscle angiogenesis and redox balance.

The present study showed that volume-dependent aerobic ET provokes changes in miRNAs-27a and -27b levels alongside ACE1/Ang II/VEGF activation, which leads to skeletal muscle angiogenesis and redox balance. Moreover, losartan treatment demonstrated the participation of RAS in ET-induced vascular growth (Figure 7). miRNAs and RAS components may be utilized as a potential target to modulate angiogenesis for combating vascular diseases. Moreover, they represent promising potential biomarkers for monitoring training interventions and physical performance.

## Data Availability

Data is contained within the article.
